# Clinical Benefits of Octreotide for Managing Persistent Gastrointestinal Fluid Loss in Malignant Small Bowel Obstruction: A Case Report and Literature Review

**DOI:** 10.7759/cureus.66785

**Published:** 2024-08-13

**Authors:** Chalothorn Wannaphut, Landon A Kozai, Narathorn Kulthamrongsri, Chutawat Kookanok, Wanprapit Noree, Kamonluk Rodsom, Ekamol Tantisattamo, Jared Acoba

**Affiliations:** 1 Department of Internal Medicine, University of Hawai'i John A. Burns School of Medicine, Honolulu, USA; 2 Department of Medicine, American Heart Association Comprehensive Hypertension Center, University of California Irvine Medical Center, Orange, USA; 3 Division of Nephrology, Hypertension and Kidney Transplantation, University of California Irvine School of Medicine, Orange, USA; 4 Department of Internal Medicine, Phramongkutklao College of Medicine, Mahidol University, Bangkok, THA; 5 Department of Internal Medicine, Faculty of Medicine Siriraj Hospital, Mahidol University, Bangkok, THA; 6 Department of Hematology and Oncology, The Queen's Medical Center, Honolulu, USA

**Keywords:** gastrointestinal fluid loss, acute kidney injury, metabolic alkalosis, octreotide, malignant small bowel obstruction

## Abstract

Malignant small bowel obstruction (mSBO) is a frequent complication in patients with gastrointestinal or gynecologic cancers. For those with inoperable cancers and persistent obstructive symptoms, symptom palliation with a percutaneous gastrostomy tube (PGT) may be required. However, excessive fluid loss from the PGT can lead to significant fluid, electrolyte, and acid-base imbalances. We present a case of a man with metastatic colonic adenocarcinoma who developed mSBO, acute kidney injury, and metabolic alkalosis, all of which were effectively managed with octreotide.

## Introduction

Malignant small bowel obstruction (mSBO) frequently occurs in advanced gastrointestinal or gynecologic cancers, with an estimated global prevalence of 10-29% in colon cancer patients [1]. This condition can result from mechanical obstruction due to the tumor or functional abnormalities affecting bowel transit [1]. mSBO often indicates a poor prognosis, especially when the obstruction is deemed inoperable. Although spontaneous resolution occurs in up to 36% of cases, recurrence rates exceed 60% [1]. For patients with inoperable cancers and persistent, severe obstructive symptoms, symptom palliation with a percutaneous gastrostomy tube (PGT) may be necessary. However, PGT-related loss of gastric juices can lead to metabolic alkalosis, acute kidney injury (AKI), and electrolyte imbalances, which can be life-threatening in severe cases. We report a case of metastatic colonic adenocarcinoma complicated by mSBO, severe metabolic alkalosis, and AKI. Treatment involved IV normal saline and subcutaneous octreotide.

## Case presentation

A 64-year-old Caucasian man presented with syncope, fatigue, and generalized weakness. One year prior, he had been diagnosed with metastatic adenocarcinoma of the colon, complicated by colonic perforation. He underwent a sigmoidectomy with end colostomy creation. During surgery, a residual mesenteric tumor, adherent to the omentum, was left due to its location. Subsequently, he developed mSBO, which necessitated the placement of a venting PGT.

In the weeks leading up to his presentation, he experienced a significant increase in PGT output, reaching six to seven liters daily. This escalation led to worsening prerenal AKI and required substantial adjustments to his IV fluids. He was receiving a continuous lactated Ringer’s infusion at 120 mL/hr for 18 hours, along with a concurrent infusion of total parenteral nutrition at 2.1 L/day.

Physical examination revealed orthostatic hypotension, with blood pressure readings of 126/68 mmHg while supine and 100/56 mmHg after standing for one minute. His respiratory rate was 20 breaths per minute, and his SpO2 was 96% on room air. He appeared cachectic, and the oral exam showed tacky mucous membranes. His colostomy pouch and PGT drained greenish gastric contents. Laboratory workup indicated a blood urea nitrogen of 64 mg/dL, serum creatinine of 4.1 mg/dL, sodium of 133 mEq/L, potassium of 3.1 mEq/L, chloride of 69 mEq/L, and bicarbonate greater than 50 mEq/L. Venous blood gas analysis revealed a pH of 7.57 and a PaCO2 of 68 mmHg. Urinalysis showed a pH of 8.5, a specific gravity of 1.012, and negative ketones; otherwise, it was unremarkable. Urine chloride was 20 mmol/L (Table 1).

**Table 1:** Laboratory data comparison: two months before admission, upon hospital admission, and at hospital discharge BUN, blood urea nitrogen; Cr, creatinine; HB, hemoglobin; Hct, hematocrit; Plt, platelet

Lab	Two months before admission	Hospital admission	Hospital discharge	Reference range
Complete blood count				
WBC (10^3^/uL)	5.79	6.34	7.26	3.8-10.84
Hb (g/dl)	8.4	9.5	8.2	13.7-17.5
Hct (%)	25	28	25.3	40.1-51.0
Plt (10^3^/uL)	133	219	175	151-424
Biochemical findings				
BUN (gm/dL)	32	64	28	6.0-23
Cr (gm/dL)	1.3	4.1	1.4	0.6-1.4
Na (mEq/L)	141	133	138	133-145
K (mEq/L)	3.6	3.1	4	3.3-5.1
Cl (mEq/L)	109	69	101	95-108
HCO3 (mEq/L)	24	>50	22	21-30
Ca (mEq/L)	8.7	9.8	8.9	8.3-10.5
Mg (mEq/L)	2.2	2.8	1.9	1.6-2.6
Phosphate (mEq/L)	3.4	4.7	3	2.5-4.5
Urinalysis				
pH	5.5	8.5	5.5	5.0-7.5
Specific gravity	1.018	1.012	1.019	1.005-1.030
Ketone	Negative	Negative	Negative	Negative
Na	-	25		
K	-	8.4		
Chloride	-	20		

The patient initially received IV normal saline and pantoprazole to restore intravascular volume and reduce gastric secretions. Despite increasing hourly saline infusions, PGT output continued to rise. A trial of clonidine patch 0.1 mg applied over 24 hours for five days also failed to provide an adequate therapeutic response. During this period, serial laboratory analyses revealed persistent AKI and metabolic alkalosis.

Given the ongoing difficulties, the decision was made to initiate subcutaneous octreotide administration at a dose of 200 mcg three times daily. Within three days, the G tube output decreased from 7 liters to 4 liters per day and was further reduced to 2 liters per day after two weeks of treatment. No short-term adverse effects were observed. Both the AKI and metabolic alkalosis resolved, and the patient was discharged with outpatient follow-up. He was placed on maintenance therapy with long-acting subcutaneous octreotide at 30 mg every four weeks.

## Discussion

The patient presented with syncope resulting from orthostatic hypotension, which was linked to severe volume depletion caused by mSBO. This condition led to hypochloremic, hypokalemic metabolic alkalosis due to substantial gastric acid and fluid losses through the PGT. Despite a bicarbonate level of 63 mEq/L and a pH of 7.57, the patient showed minimal symptoms. The syncopal event was likely attributed to orthostatic hypotension rather than alkalemia. Profound metabolic alkalosis can result in serious complications such as cardiopulmonary arrest, stupor, coma, dysrhythmia, and seizures [2,3].

In this case, metabolic alkalosis arises from the loss of hydrogen (H⁺) and chloride (Cl⁻) ions due to gastric parietal cell dysfunction. Carbonic anhydrase mediates this process, producing bicarbonate (HCO₃⁻), which is normally excreted by the kidneys. Volume depletion triggers the renin-angiotensin-aldosterone system, leading to secondary hyperaldosteronism. The actions of aldosterone and angiotensin II result in renal potassium wasting. Combined with hypochloremia and reduced kidney perfusion, these factors contribute to renal tubular mechanisms that sustain metabolic alkalosis [4].

This case was notable for its unusually high gastric fluid losses, which were exacerbated by escalating IV crystalloid infusions and only alleviated with the addition of octreotide. While gastric fluid loss is a recognized cause of metabolic alkalosis, reports of daily fluid losses reaching six to seven liters are rare. In mSBO, increased intraluminal fluids and gas lead to bowel distension and heightened peristalsis. This triggers the release of 5-HT3 from enterochromaffin cells, which activates the enteric nervous system through mediators such as prostaglandins and vasoactive intestinal peptide (VIP). This process results in excessive water and electrolyte secretion by intestinal crypts, contributing to severe edema and increased intraluminal pressure [1].

The clinical manifestations of mSBO typically include severe nausea, vomiting, colicky pain, and abdominal distension. Partial obstructions may lead to high-volume liquid stools due to increased intestinal hypersecretion. Octreotide is the treatment of choice for reducing symptoms and hypersecretion. For persistent symptoms, options include nasogastric tube (NGT) placement or venting gastrostomy. Surgical interventions such as diverting ileostomies may result in high-volume output and may need to be reversed once secretions are significantly reduced. High-volume output from a PGT is a common complication in inoperable mSBO cases. Despite extensive IV crystalloid resuscitation, persistent and worsening intestinal hypersecretion can exacerbate AKI and electrolyte imbalances. Therefore, early initiation of octreotide therapy is crucial for managing symptoms and reducing fluid secretion in patients with high-volume PGT output.

Octreotide, developed as a somatostatin analog, is an inhibitory hormone that suppresses the release of various hypothalamic hormones, including growth hormone, thyroid-stimulating hormone, prolactin, and adrenocorticotropic hormone. It also inhibits insulin secretion and various peptides in the gastroenteropancreatic system, reducing blood flow in the splanchnic and portal systems. Consequently, it affects gastrointestinal functions by modulating motility and secretion in the stomach, pancreas, and small bowel, while enhancing water and electrolyte absorption [5].

By mimicking somatostatin, octreotide binds to receptors in the pancreas and small intestine, thereby reducing the secretion of water, sodium, and chloride. Its antisecretory action inhibits VIP, which leads to decreased hydroelectrolytic retention, reduced gastric secretions, diminished intestinal motility, lower biliary flow, and reduced splanchnic hypervascularization and parietal edema. These effects make octreotide an effective treatment for conditions associated with excessive gastrointestinal secretions, including bowel obstruction [1].

The optimal dose of octreotide has not been firmly established, but clinical reports suggest that while up to 50% of patients may respond to the typical starting dose of 300 micrograms per 24 hours, a higher response rate of 75-90% is seen with doses of 600-800 micrograms per 24 hours. Although doses up to 1,500 micrograms per 24 hours have been used, 600-800 micrograms per 24 hours is generally adequate to determine likely responders [5]. Daily doses ranging from 300 to 600 micrograms have shown good symptomatic outcomes [6]. Despite the limited number of controlled clinical trials, octreotide remains the preferred antisecretory agent for treating bowel obstruction. The effectiveness of octreotide in managing mSBO in our case aligns with these findings.

In a prospective study involving ovarian cancer patients with mSBO, the administration of 300-600 micrograms of octreotide resulted in complete symptom relief within three days (range: one to six days). Most patients experienced cessation of vomiting within two to three days of initiating treatment. Additionally, octreotide reduced NGT drainage in eight patients, allowing for its removal without adverse effects. Patients continued octreotide therapy at home and had an average survival time of 15 days [7].

In another trial involving 11 mSBO patients, a home-administered regimen combining octreotide (0.3 mg/24 h), metoclopramide (1 mg/kg/24 h), and dexamethasone (16 mg/day IV bolus) demonstrated safety and rapid relief of gastrointestinal symptoms. Patients achieved regular bowel movements within five days, indicating that this combination therapy may be a promising option for managing mSBO-related symptoms [8].

Octreotide is established as an effective treatment for reducing gastrointestinal secretions, colic, nausea, and vomiting associated with mSBO. This is supported by one systematic review and three randomized trials. In a comparative study, scopolamine butylbromide, when administered in equivalent doses, was found to be less effective than octreotide [9].

In addition to octreotide, alternative medications for managing secretory diarrhea - such as clonidine and crofelemer - may be considered. Clonidine is effective in treating high gastrointestinal fluid loss, which can occur in conditions like short bowel syndrome (SBS) with elevated intestinal output. This case report suggests that clonidine might be a valuable adjunct therapy for patients with SBS and high intestinal outputs that are unresponsive to conventional treatments [6]. In a study with healthy volunteers, clonidine reduced diarrhea volume by 48% following the ingestion of an oral electrolyte solution [10]. However, its effects on intestinal motility remain uncertain due to conflicting results. Some studies indicated that clonidine (0.3 mg) prolonged small intestinal transit time as measured by the hydrogen breath test [11,12], while others found no impact on gastric emptying or small-bowel transit time using radiolabeled markers [13,14].

Crofelemer, a novel agent, effectively manages secretory diarrhea, including traveler’s diarrhea, AIDS-associated diarrhea, and diarrhea-predominant irritable bowel syndrome. It works by blocking two intestinal chloride channels, thereby preventing chloride and fluid secretion into the intestine [5,15].

We report a case demonstrating the efficacy of octreotide in treating prerenal AKI and metabolic alkalosis associated with mSBO. Several studies have highlighted the effectiveness of alternative medications, such as clonidine and crofelemer, as well as combination regimens involving octreotide, metoclopramide, and dexamethasone, for managing inoperable mSBO cases. These findings underscore the importance of considering these medications for nonsurgical approaches to mSBO management. Based on the current literature, clinicians should consider octreotide as a viable option for nonsurgical management of mSBO. Nevertheless, further comprehensive research is needed to definitively establish the efficacy and safety of these combination therapies.

## Conclusions

Our case underscores the challenge of severe volume depletion due to mSBO, which led to syncope from orthostatic hypotension. The associated hypochloremic, hypokalemic metabolic alkalosis, despite its potential severity, was minimally symptomatic. Octreotide proved crucial in managing the unusually high gastric fluid losses, offering an effective nonsurgical intervention to alleviate metabolic alkalosis and control mSBO-related symptoms.

Additionally, alternative medications such as clonidine and crofelemer, along with combination regimens involving octreotide, metoclopramide, and dexamethasone, show promise for managing inoperable mSBO cases. Although octreotide is a viable nonsurgical treatment option, further research is needed to definitively establish the efficacy and safety of these therapeutic approaches. Clinicians should carefully evaluate the available evidence and tailor treatment plans to individual patient needs to optimize outcomes in managing mSBO.
